# Supplementary material for: Computational modeling of threat learning reveals links with anxiety and neuroanatomy in humans
Source: eLife. 2022 Apr 27;11:e66169. doi: 10.7554/eLife.66169 (PMC9197395; doi:10.7554/eLife.66169)
Supplement: Source code 1. [file elife-66169-code1.zip › modeling_code.docx]

%% These functions fit the 9 models (8 versions of Rescorla-Wagner(RW) and hybrid Pearce-Hall(PH) to SCR data for the paper “Computational Modeling of Threat Learning Reveals Links with Anxiety and Neuroanatomy in Humans” by Abend et al.

% Below is an example of how one would fit 8 RW models to a dataset:

% For the 8 RW models, a matrix was used to define the presence of

% additional model features. A 1 indicates that the model feature is

% included and a 0 indicates that it is not included

% Column 1 = habituation params

% Column 2 = bayesian learning rate

% Column 3 = learning inertia

% model = [0 0 0;

% 0 0 1;

% 0 1 0;

% 1 0 0;

% 0 1 1;

% 1 0 1;

% 1 1 0;

% 1 1 1];

% A single model was chosen for each run of the fit. The function fitSCRGen

% takes the model information as an input (e.g.: modelinfo=model(1,:) would

% be the simplest RW model, model 1)

% Then use fminsearch to minimize the negative log-likelihood of the fit to

% the model (set options for limits on fminsearch):

% [fitparams, ll] = fminsearch(@(params) fitSCRGen(params, scr, us, csType, vi, modelinfo, n, mu), params, options);

%inputs:

%params: starting values for parameters

%scr: skin conductance response to conditioned stimulus

%us: skin conductance response to unconditioned stimulus

%vi: initial value estimate

%modelinfo: 3 element vector defining which model should be fit

%outputs:

%fitparams: values of the parameters from the particular fit

%ll: log likelihood of fit

%% %%%%%%%%%%%%%%%%%%%%%%%%%%%%%%%%%%%%%%%%%%%%%%%%%%%%%%%%%%%%%%%%%%%%%%%%%

%%%%%%% Model Functions %%%%%%%%%%%%%%

%%%%%%%%%%%%%%%%%%%%%%%%%%%%%%%%%%%%%%%%%%%%%%%%%%%%%%%%%%%%%%%%%%%%%%%%%%%

%Wrapper function for fitSCRfGen, for compatibility with fminsearch :

function [ll] = fitSCRGen(params, scr, us, csType, vi, modelV, n, mu)

[ll, ~] = fitSCRfGen(params, scr, us, csType, vi, modelV, n, mu);

end

%%%%%%%%%%%%%%%%%%%%%%%%%%%%%%%%%%%%%%%%%%%%%%%%%%%%%%%%%%%%%%%%%%%%%%%%%%%

%For fitting 8 RW models to acquisition data:

function [ll, v] = fitSCRfGen(params, scr, us, csType, vi, modelV, n, mu)

v = zeros(2, length(scr) + 1);

v(1:2, 1) = vi;

rpe = zeros(length(scr), 1);

for trial = 1 : length(scr)

rpe(trial) = us(trial) - v(csType(trial), trial);

if modelV(2) == 1

lrp1 = params(1)/(trial^1/2);

lrp2 = params(2)/(trial^1/2);

else

lrp1 = params(1);

lrp2 = params(2);

end

if modelV(3) == 1

ftrial = min(trial-1,2);

if trial > 1

srpe = sum(rpe(trial:-1:trial-ftrial));

else

srpe = rpe(trial);

end

else

srpe = rpe(trial);

end

if csType(trial) == 1

v(1, trial+1) = v(1, trial) + lrp1*srpe + mu(trial);

v(2, trial+1) = v(2, trial) + lrp2*srpe + mu(trial);

else

v(1, trial+1) = v(1, trial) + mu(trial);

v(2, trial+1) = v(2, trial) + mu(trial);

end

end

z1 = find(csType == 1);

z2 = find(csType == 2);

if modelV(1) == 1

exp1 = params(3);

exp2 = params(4);

if isnan(n)

vec = [0 0];

n = 2;

else

vec = zeros(n,1)';

end

v(1, z1) = v(1, z1).*exp(-exp1*([vec 1:(length(z1) - n)]));

v(2, z2) = v(2, z2).*exp(-exp2*([vec 1:(length(z2) - n)]));

end

ll = 0;

for trial = 1 : length(scr)

ll = ll + abs(v(csType(trial), trial) - scr(trial)).^2;

end

end

%%%%%%%%%%%%%%%%%%%%%%%%%%%%%%%%%%%%%%%%%%%%%%%%%%%%%%%%%%%%%%%%%%%%%%%%%%%

%Wrapper function for fitSCRfGenExt, for compatibility with fminsearch :

function [ll] = fitSCRGenExt(params, scr, us, csType, vi, modelV, mu)

[ll, ~] = fitSCRfGenExt(params, scr, us, csType, vi, modelV, mu);

end

%%%%%%%%%%%%%%%%%%%%%%%%%%%%%%%%%%%%%%%%%%%%%%%%%%%%%%%%%%%%%%%%%%%%%%%%%%%

%For fitting 8 RW models to extinction data:

function [ll, v] = fitSCRfGenExt(params, scr, us, csType, vi, modelV, mu)

v = zeros(2, length(scr) + 1);

v(1:2, 1) = vi;

rpe = zeros(length(scr), 1);

for trial = 1 : length(scr)

rpe(trial) = us(trial) - v(csType(trial), trial);

if modelV(2) == 1

lrp1 = params(1)/(trial^1/2);

lrp2 = params(2)/(trial^1/2);

else

lrp1 = params(1);

lrp2 = params(2);

end

if modelV(3) == 1

ftrial = min(trial-1,1);

if trial > 1

srpe = sum(rpe(trial:-1:trial-ftrial));

else

srpe = rpe(trial);

end

else

srpe = rpe(trial);

end

csn_seq = find(csType == 2);

first_csn = csn_seq(1);

if trial < first_csn || first_csn == 1

if csType(trial) == 1

v(1, trial+1) = v(1, trial) + lrp1*srpe + mu(trial);

v(2, trial+1) = v(2, trial) + mu(trial);

else

v(1, trial+1) = v(1, trial) + mu(trial);

v(2, trial+1) = v(2, trial) + lrp2*srpe + mu(trial);

end

else

if csType(trial) == 1

v(1, trial+1) = v(1, trial) + lrp1*srpe + mu(trial);

v(2, trial+1) = v(2, trial) + lrp2*srpe + mu(trial);

else

v(1, trial+1) = v(1, trial) + mu(trial);

v(2, trial+1) = v(2, trial) + mu(trial);

end

end

end

z1 = find(csType == 1);

z2 = find(csType == 2);

if modelV(1) == 1

exp1 = params(3);

exp2 = params(4);

v(1, z1) = v(1, z1).*exp(-exp1*([0 1:(length(z1)-1)]));

v(2, z2) = v(2, z2).*exp(-exp2*([0 1:(length(z2)-1)]));

end

ll = 0;

for trial = 1 : length(scr)

ll = ll + abs(v(csType(trial), trial) - scr(trial)).^2;

end

end

%%%%%%%%%%%%%%%%%%%%%%%%%%%%%%%%%%%%%%%%%%%%%%%%%%%%%%%%%%%%%%%%%%%%%%%%%%%

%For fitting Pearce-Hall model to data:

function [ll, v] = fitSCRfPHG(params, scr, us, csType, vi, mu)

v(1:2,1) = vi;

alphpos = 1;

alphneg = 1;

for trial = 1 : length(scr)

rpe = us(trial) - v(csType(trial), trial);

if csType(trial) == 1

v(1, trial+1) = v(1, trial) + params(1)*alphpos*(rpe) + mu(trial);

v(2, trial+1) = v(2, trial) + params(2)*alphneg*(rpe) + mu(trial);

alphpos = (1-params(3))*alphpos + abs(rpe)*params(3);

alphneg = (1-params(4))*alphneg + abs(rpe)*params(4);

else

v(1, trial+1) = v(1, trial) + mu(trial);

v(2, trial+1) = v(2, trial) + mu(trial);

end

end

ll = 0;

for trial = 1 : length(scr)

ll = ll + abs(v(csType(trial), trial) - scr(trial)).^2;

end

end
