# Supplementary material for: Computational modeling of threat learning reveals links with anxiety and neuroanatomy in humans
Source: eLife. 2022 Apr 27;11:e66169. doi: 10.7554/eLife.66169 (PMC9197395; doi:10.7554/eLife.66169)
Supplement: Source code 2. [file elife-66169-code2.zip › imaging_code.docx]

%% Analysis pipeline for imaging data for the paper “Computational Modeling of Threat Learning Reveals Links with Anxiety and Neuroanatomy in Humans” by Abend et al.

1. Run FreeSurfer for each participant to align, register, and segment the brain images:

recon-all -subjid subj -i output/subj.nii.gz -all

2. Removal of poor surface reconstructions after visual inspection using procedure described in: https://brainder.org/2011/09/10/quickly-inspect-freesurfer-cortical-surfaces/

3. Run mris_preproc to smooth and put all participants in the same common grid for between-subject comparisons:

e.g.: mris_preproc subj --hemi rh --meas thickness –out rh.thickness.mrispreproc.mgh --nocleanup --fwhm-src 20 --target fsaverage5

4. Merge hemispheres to allow multiple testing correction across both hemispheres (bh):

palm_hemimerge lh* rh* (documentation: https://github.com/andersonwinkler/PALM/blob/master/palm_hemimerge.m)

5. Extract volumes of subcortical structures from the outputs of FreeSurfer:

asegstats2table -s subj

6. Compute the amount of gray matter within each subcortical structure, using asegpve, which in turn uses FSL FAST for segmentation into GM/WM/CSF:

asegpve -s subj (documentation: https://github.com/andersonwinkler/toolbox/blob/master/bin/asegpve)

7. Prepare design matrices and contrast files for PALM analysis. While this could have been accomplished manually, a MATLAB script was created:

% Load variables data: ID, age, anxiety, sex, DV

D = strcsvread(‘variables_data.csv'),',');

% Get the DV data (e.g., model parameter), save

PARAM = cell2mat(D(2:end,strcmp(D(1,:),'param')));

csvwrite(fullfile(palmdir,'param.csv'), PARAM);

% Get age and center

age = cell2mat(D(2:end,strcmp(D(1,:),'age')));

age = bsxfun(@minus,age,mean(age));

% Get anxiety scores and center

anxiety = D(2:end,strcmp(D(1,:),' anxiety'));

anxiety = cell2mat(anxiety);

anxiety = bsxfun(@minus, anxiety,mean(anxiety));

% Get the sex and center

sex = D(2:end,strcmp(D(1,:),'sex'));

idx = strcmp(sex,'Male'); sex(idx) = {1};

idx = strcmp(sex,'Female'); sex(idx) = {0};

sex = cell2mat(sex);

sex = bsxfun(@minus,sex,mean(sex));

% Create an intercept and a dummy

I = ones(size(age));

dummy = I*9999;

% Load the global variables (global thickness, ICV)

G = strcsvread('globals.csv'),'\t');

Globals = cell(2,1);

Globals{1} = cell2mat(G(2:end,strcmp(G(1,:),'bh.MeanThickness')));

Globals{2} = cell2mat(G(2:end,strcmp(G(1,:),'EstimatedTotalIntraCranialVol')))./1e6;

Global_names = {'globalthickness','globalicv'};

% Subcortical imaging data:

img = load(‘gmv_data.csv');

csvwrite(‘gmv.csv', img);

csvwrite(‘gmv.age.csv', img.*age);

csvwrite(‘gmv.anx.csv', img.*anx);

csvwrite(‘gmv.age.anx.csv', img.*age.*anx);

mask = ones(1,size(img,2));

csvwrite('mask.csv'),mask);

% Cortical imaging data:

x = palm_miscread('bh.thickness.mrispreproc.mgz');

img = permute(x.data,[4 1 2 3]);

x.data = permute(img,[2 3 4 1]); x.filename = 'bh.thickness'; palm_miscwrite(x,false);

x.data = permute(img.*age,[2 3 4 1]); x.filename = 'bh.thickness.age'; palm_miscwrite(x,false);

x.data = permute(img.*dx,[2 3 4 1]); x.filename = 'bh.thickness.dx'; palm_miscwrite(x,false);

x.data = permute(img.*age.*dx,[2 3 4 1]); x.filename = 'bh.thickness.age.dx'; palm_miscwrite(x,false);

% Compute the average area per vertex, to be used for spatial statistics

x = palm_miscread('bh.area.mgz');

x.data = mean(x.data,4);

x.filename = 'bh.avg_area_per_vertex';

palm_miscwrite(x,false);

% Create the designs; dummies are replaced by img*age, for example, to predict DV

M = [I ... % intercept (1)

dummy ... % imaging data (2)

age ... % age (3)

anx ... % anxiety (4)

sex ... % sex (5)

age.*anx ... % age*anx (6)

dummy ... % img*age (7)

dummy ... % img*anx (8)

dummy]; % img*age*anx (9)

for g = 1:numel(Globals) csvwrite('design_incl_global_%s.csv',Global_names{g})),horzcat(M,Globals{g}))

end

% Create the contrast files

C = [...

0 0 0 0 0 0 0 0 +1;

0 0 0 0 0 0 0 0 -1;

0 0 0 0 0 0 0 +1 0;

0 0 0 0 0 0 0 -1 0;

0 0 0 0 0 0 +1 0 0;

0 0 0 0 0 0 -1 0 0;

0 +1 0 0 0 0 0 0 0;

0 -1 0 0 0 0 0 0 0];

C = horzcat(C,zeros(size(C,1),1));

csvwrite('contrasts_incl_global.csv',C);

8. Run PALM (documentation: https://fsl.fmrib.ox.ac.uk/fsl/fslwiki/PALM):

PALM -i predicted_var.csv -d design_incl_global_globalthickness.csv -t contrasts_incl_global.csv -evperdat bh.thickness.mgz 2 1 -evperdat bh.thickness.age.mgz 7 1 -evperdat bh.thickness.anx.mgz 8 1 -evperdat bh.thickness.age.anx.mgz 9 1 -m bh.FS.ic5.aparc.mask.dpv -s bh.white.srf bh.avg_area_per_vertex.mgz -T -designperinput -logp -nouncorrected -approx tail -n 1000
